# Protein Oxidative Modifications in Neurodegenerative Diseases: From Advances in Detection and Modelling to Their Use as Disease Biomarkers

**DOI:** 10.3390/antiox13060681

**Published:** 2024-05-31

**Authors:** Sandra I. Anjo, Zhicheng He, Zohaib Hussain, Aruba Farooq, Alan McIntyre, Charles A. Laughton, Andreia Neves Carvalho, Mattéa J. Finelli

**Affiliations:** 1 CNC-Center for Neurosciences and Cell Biology, University of Coimbra, 3004-517 Coimbra, Portugal; 2 Centre for Innovative Biomedicine and Biotechnology (CIBB), University of Coimbra, 3004-517 Coimbra, Portugal; 3 Institute for Interdisciplinary Research (IIIUC), University of Coimbra, 3030-789 Coimbra, Portugal; 4 Biodiscovery Institute, School of Pharmacy, University of Nottingham, Nottingham NG7 2RD, UK; 5 Biodiscovery Institute, School of Medicine, University of Nottingham, Nottingham NG7 2RD, UK; 6 Research Institute for Medicines (iMed.ULisboa), Faculty of Pharmacy, Universidade de Lisboa, 1649-003 Lisbon, Portugal; 7 Department of Pharmaceutical Sciences and Medicines, Faculty of Pharmacy, Universidade de Lisboa, 1649-003 Lisbon, Portugal

**Keywords:** redox post-translational modifications, neurodegenerative diseases, Alzheimer’s disease, Parkinson’s disease, redox proteomics, computational modelling, molecular dynamics, biomarkers

## Abstract

Oxidation–reduction post-translational modifications (redox-PTMs) are chemical alterations to amino acids of proteins. Redox-PTMs participate in the regulation of protein conformation, localization and function, acting as signalling effectors that impact many essential biochemical processes in the cells. Crucially, the dysregulation of redox-PTMs of proteins has been implicated in the pathophysiology of numerous human diseases, including neurodegenerative diseases such as Alzheimer’s disease and Parkinson’s disease. This review aims to highlight the current gaps in knowledge in the field of redox-PTMs biology and to explore new methodological advances in proteomics and computational modelling that will pave the way for a better understanding of the role and therapeutic potential of redox-PTMs of proteins in neurodegenerative diseases. Here, we summarize the main types of redox-PTMs of proteins while providing examples of their occurrence in neurodegenerative diseases and an overview of the state-of-the-art methods used for their detection. We explore the potential of novel computational modelling approaches as essential tools to obtain insights into the precise role of redox-PTMs in regulating protein structure and function. We also discuss the complex crosstalk between various PTMs that occur in living cells. Finally, we argue that redox-PTMs of proteins could be used in the future as diagnosis and prognosis biomarkers for neurodegenerative diseases.

## 1. Introduction

Oxidation–reduction post-translational modifications (redox-PTMs) are oxidative modifications that consist of redox-based chemical alterations occurring on the amino acids of proteins in the presence of reactive oxygen species (ROS) and/or reactive nitrogen species (RNS) [1]. These redox-PTMs play essential roles in the cellular response to stress conditions and maintenance of redox homeostasis and, more generally, in cellular signalling pathways. Redox-PTMs are thus fundamental both in physiological conditions and under chronic oxidative stress, as observed in neurodegenerative diseases (NDDs), such as Alzheimer’s disease (AD), Parkinson’s disease (PD), Amyotrophic Lateral Sclerosis (ALS), Frontotemporal Dementia (FTD), Dementia with Lewy Bodies (DLB), and neuroinflammatory disease Multiple Sclerosis (MS) [2,3,4,5,6,7,8]. Indeed, redox-PTMs are essential for the integration and transformation of oxidant signals into biological responses through redox-dependent intracellular signalling pathways, a topic that has been reviewed elsewhere [9,10,11]. Redox-PTMs can modulate protein conformation, and, in the context of NDDs where aberrant redox-PTMs can be present, this may lead to protein misfolding, mislocalisation, and aggregation [8,12,13,14].

In this review, we aim to highlight the current gaps in knowledge in the field of redox-PTMs biology. We will first briefly summarize the main types of redox-PTMs in mammalian cells, providing examples of proteins modified by redox-PTMs in NDDs, and give an overview of the state-of-the-art methods used in redox-PTMs detection. We will explore how new computational modelling strategies have the potential to help better understand the role of redox-PTMs in modulating protein structure and function. We will also explore the importance of the complex, but still poorly understood, process of PTMs crosstalk in modulating protein function. Finally, we will discuss whether redox-PTMs of proteins could be used in future clinical studies as biomarkers to help with the diagnosis and prognosis of NDDs such as AD and PD.

## 2. Oxidative Modifications

**Figure 1:**
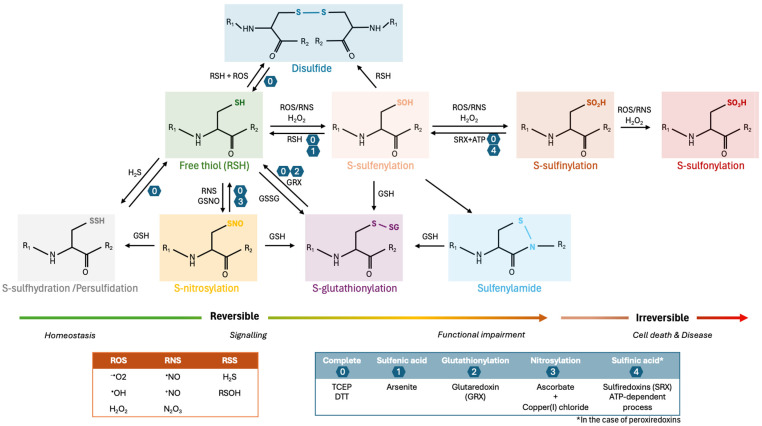
Different types of redox-PTMs occurring in Cysteine residues of proteins (oxoforms), their interchangeability and reversibility. Cysteines (Cys) can undergo oxidation forming different oxidized species, which can be converted into other oxoforms. Generally, there are two primary levels of redox-PTMs on Cys residues of proteins based on cellular oxidative stress levels: reversible and irreversible Cys oxidations, as indicated by the coloured arrows. Reversible oxidation of Cys, such as S-nitrosylation (S-NO), S-sulfenylation (S-OH), S-glutathionylation (S-SG), and disulphide formation (S-S), occurs under low oxidative conditions and modulates protein function. At higher levels of oxidants (indicated in the orange box) or prolonged exposure to oxidative stress, the oxidative state of Cys becomes elevated and more difficult to reverse (S-sulfinylation, S-O_2_H) or irreversible (S-sulfonylation (S-O_3_H) or S-sulfinylation (S-O_2_H) for all proteins except peroxiredoxins). These over-oxidized forms lead to loss of protein function and are commonly associated with cell death and disease pathophysiology. The blue box summarizes the chemical and enzymatical processes that can be used to experimentally reduce Cys. Adapted from [35,36,37,38]. TCEP: tris(2-carboxyethyl)phosphine; DTT: dithiothreitol; ROS: reactive oxygen species.

Due to the high reactivity of certain amino acids, proteins are primary targets for redox reactions [3] directly with ROS and RNS as well as with highly reactive intermediates formed during the degradation of lipids and carbohydrates as a result of ROS/RNS exposure [15]. A wide range of reversible and irreversible (i.e., PTMs that cannot be reduced under physiological conditions) redox-PTMs of proteins can be generated by oxidation of the side chains of sulphur-containing amino acids (cysteine and methionine) and of aromatic residues (tryptophan, tyrosine, phenylalanine, and histidine) [15,16,17]. Importantly, although cysteine residues (Cys) have relatively low abundance (representing only approximately 3.3% of the human proteome [18,19,20,21]), they are one of the most commonly redox-modified amino acid residues due to the high reactivity of their sulphur-based functional group and can be modified by several redox-PTMs (Figure 1) [3,20,22,23].

### 2.1. Sulfenic, Sulfinic and Sulfonic Acids

S-Sulfenylation is the oxidation of Cys thiol groups by ROS such as the anion radical superoxide and hydrogen peroxide, which leads to Cys residues carrying sulfenic acid (-SOH) groups [24,25,26]. Due to the rapid conversion of sulfenic acid to other species, this modification is considered an essential regulatory mechanism in redox signalling, acting as a transient intermediary to further reversible (e.g., S-glutathionylation) or irreversible (e.g., S-sulfonation) modifications (Figure 1) [24,26]. Usually, the over-oxidation of thiols, which leads to the formation of sulfinic (-SO_2_H) and sulfonic (-SO_3_H) acids, occurs under oxidative stress conditions and is often associated with functional impairment of proteins and cell death [27]. Hence, sulfenic acid formation is a determinant step in cell fate decisions towards damage or survival. Interestingly, an increase in protein S-sulfenylation and S-sulfonylation has been observed with ageing and might contribute to conformational changes that increase the propensity for protein aggregation. Among the proteins altered are synapsin-1 and Ras-GTPase Activating Protein SH3 Domain-Binding Protein 2 (G3BP2), resulting in disruptions in neurotransmitter release and stress granule formation, respectively [28].

The formation of sulfinic acids (-SO_2_H) has long been considered to be irreversible; however, this has lately been challenged by the discovery of Peroxiredoxin (Prdx) and Sulfiredoxin (Srx) enzymes. Prdxs are reversibly inactivated by oxidation of the catalytic Cys residue to sulfinic acid in cells, while Srx enzymes have sulfinic acid-reductase activity capable of reverting the formation of SO_2_H [29,30,31,32]. Prdx2, the most abundant Peroxiredoxin in neurons, has a protective effect against oxidative stress, but its enzymatic activity is itself inhibited by S-nitrosylation of the active site residues Cys51 and Cys172 [33], with this specific modification shown to be increased in human postmortem PD brains [33]. This example illustrates how crosstalk between different redox-PTMs is relevant in oxidative signalling regulation, as will be discussed in further detail in Section 5.1. Members of the thiol-based Prdx family of enzymes act as antioxidants and chaperones regulating redox-signalling and therefore impact numerous physiological and pathological mechanisms [34]. Furthermore, the Prx/Srx system is believed to function to enable localized oxidative signalling.

### 2.2. Disulfide Bonds

Disulfide bonds can be formed between two independent free thiols/thiyl radicals (-S•) that, upon oxidation, establish a covalent linkage between them (-S-S-). Disulfide bonds can also be formed through the reaction of a sulfenic acid (-SOH), which is converted to a thiyl radical in the presence of oxidative stress and reacts with other thiolates. These modifications can occur within a protein (intramolecular covalent bonds), or between proteins (intermolecular covalent bonds) and therefore are structurally fundamental as they stabilize tertiary and quaternary protein structures [39]. The formation of disulfide bonds in the endoplasmic reticulum (ER) is crucial to ensure protein folding and is tightly regulated by oxidoreductases, namely members of the protein disulfide isomerase (PDI) family [40,41]. Disulfide bonds are found in 15% of the human proteome, with a notable enrichment in secreted proteins (65%), attributed to the necessity for enhanced protein stability in the particularly oxidizing extracellular environment [42]. The vast majority of NDDs have been associated with misfolding and aggregation of proteins, either due to protein mutations or over-oxidation, with intermediate oligomeric species frequently being associated with cellular toxicity. Because disulfide bonds are highly conserved and crucial structural characteristics of proteins, they are likely key determinants of misfolding and aggregation of proteins in NDDs [42,43]. Interestingly, it has been described that intramolecular disulfide bonds favour stabilization of the native structure of a protein decreasing the tendency for amyloid fibrils formation, whereas intermolecular disulfide bonds facilitate aggregation [44]. For instance, an increase in disulfide bonds in glyceraldehyde 3-phosphate dehydrogenase (GAPDH), which is a redox-sensitive glycolytic enzyme, has been reported in the brain of AD patients, which in turn results in decreased enzymatic activity and increased aggregation [45]. Interestingly, GAPDH has been found to accumulate in neurofibrillary tangles, Lewy bodies and other detergent-insoluble plaques associated with NDDs, suggesting that defects in redox-PTMs such as disulfide bonds in GAPDH contribute to the pathomechanisms of NDDs [46].

### 2.3. S-Sulfhydration

The process of S-sulfhydration, also known as persulfidation or thiolation, involves the addition of hydrogen sulfide (H_2_S) to Cys residues, leading to the formation of a persulfide bond (-S-SH) [38,47]. Although H_2_S is ubiquitously present in the human organism, initial reports of its role in the central nervous system focused on the deleterious effects of exposure to high exogenous doses of H_2_S, which is considered an environmental toxin [47]. However, it is now well-established that H_2_S is present under physiological conditions and involved in important physiological functions—in particular, neurotransmission [48,49,50,51]. Recent developments in techniques of labelling and detection of persulfides have enabled the uncovering of some of the roles of this redox-PTM in (patho)physiology [47,51]. Formation of -S-SH has been suggested as a way to protect proteins from oxidative stress-induced damage and to be neuroprotective [52].

Interestingly, persulfidation has been reported to decrease with ageing, contributing to higher susceptibility of proteins to oxidative damage [51], which has been gaining particular relevance considering that ageing is the main risk factor associated with NDDs. Accordingly, altered persulfide levels have been reported in various NDDs [47]. For instance, levels of H_2_S have been shown to be altered in the brains and plasma of AD patients and animal models of AD [53,54], and cystathionine γ-lyase (CSE), the rate-limiting enzyme of H_2_S biosynthesis, has been shown to be depleted in 3xTg-AD mouse models as well as in human AD brains [55]. Additionally, H_2_S can prevent Tau hyperphosphorylation, a pathological hallmark of AD pathogenesis, by S-sulfhydration of its kinase, glycogen synthase kinase3β (GSK3β), at Cys218 [55].

The importance of persulfidation in neurodegeneration has also been highlighted in a very recent study that showed that the oxidation of Cys thiol groups to Cys sulfenylates promotes protein aggregation, which suggested that persulfidation works as a protective mechanism preventing protein aggregation and subsequent neurodegeneration [28].

### 2.4. S-Glutathionylation

S-Glutathionylation is a reversible redox-PTM that involves the addition of nucleophilic glutathione to electrophilic sulfhydryl or sulfenic acid moieties of Cys residues, which leads to the formation of mixed disulfides (Figure 1) [56]. S-glutathionylation can occur spontaneously through the direct thiol-disulfide exchange between a protein thiol and an oxidized glutathione (GSSG) molecule, leading to the formation of S-glutathionylated proteins (PSSG) [57]. To occur spontaneously, thiol-disulfide exchange requires an oxidizing environment, which is mainly observed under pathological but not physiological conditions [58,59,60]. An alternative mechanism involves the initial oxidative modification of a reduced protein thiol (e.g., thiyl radical -S•, sulfenic acid -SOH, S-nitrosylation product -SNO), which can subsequently react with a reduced glutathione (GSH) molecule [57]. This can occur through enzymatic reactions catalysed by Glutathione S-transferase pi (GSTP) or Glutaredoxins (Grx) [61,62,63]. Several reports have also established a role for Cys-rich proteins, such as Thioredoxin (Trx), Srx and Grx in the reversal glutathionylation process (deglutathionylation) [64,65,66], with Grx catalysing both the forward and reverse reactions of S-glutathionylation [63,64,67] and GSTP potentiating S-glutathionylation reactions in response to oxidative and nitrosative stress [61]. PDI has also been suggested as a putative deglutathionylation enzyme [68]. Interestingly, silencing of glutamate-cysteine ligase (GCL), the rate-limiting enzyme of glutathione biosynthesis, induced the progressive degeneration of nigral dopaminergic neurons, and overexpression of this enzyme led to aberrant S-glutathionylation of neuronal proteins [69].

Through the reversible formation of disulfides, S-glutathionylation generally functions as a ‘reservoir’ for a reduction in protein thiols during recovery from oxidative stress to restore protein functions [70]. Accordingly, S-glutathionylation has been suggested to protect Cys residues from further (and possibly irreversible) oxidation, preventing sulfhydryl overoxidation and proteolysis [58,61,63,64,71,72]. For instance, in models of PD, GSTP has been shown to protect dopaminergic neurons from oxidative stress by modulation of S-glutathionylation and stress-related cellular signalling pathways [61,73,74].

S-glutathionylation plays a crucial role in redox-signalling in response to oxidative stress acting as an adaptive cellular response and regulating the function of intracellular signalling proteins as well as transcription factors related to energy metabolism, cytoskeleton organization, calcium homeostasis, mitochondrial quality control, protein folding and apoptosis cascades [58,72,75]. The identification of proteins selectively S-glutathionylated allowed not only to provide new insights into the neuropathological consequences of S-glutathionylation in the neurodegenerative process but also to identify the S-glutathionylation of proteins as a potential biomarker for NDDs [59,63].

### 2.5. S-Nitrosylation and Nitration

S-nitrosylation and nitration are chemical processes that implicate the addition of nitrogen-containing groups to molecules; however, they operate through different mechanisms and have distinct effects and outcomes [14]. S-nitrosylation (or S-nitrosation) involves the reversible addition of nitric oxide (-NO) to proteins. S-nitrosylation can modulate the activity, localization and stability of proteins and other biomolecules by covalently modifying Cys residues, giving rise to S-nitrosothiols, which play important roles in cell signalling and regulation [8]. Increased S-nitrosylation of proteins, as observed under nitrosative stress, has been associated with the production of pathological levels of misfolded proteins [14].

Nitration leads to the irreversible addition of nitrogen dioxide (-NO_2_) to the aromatic ring of tyrosine residues of proteins [76,77]. Under oxidative stress conditions, ROS can react with NO or derived metabolites, which leads to the formation of highly reactive nitrating species, such as peroxynitrite (ONOO-). Peroxynitrite will, in turn, react with tyrosine residues, generating 3-nitrotyrosine (3-NT)-modified proteins. Nitration of tyrosine residues to 3-NT is an irreversible redox-PTM that disrupts nitric oxide signalling, shifting cell metabolism towards a more pro-oxidant status [76]. Nitration can cause structural changes in proteins, leading to their inactivation, and has been suggested to be involved in the initiation and progression of NDDs [77], such as AD and PD [78,79].

Postmortem analysis of midbrain samples has shown increased expression of neuronal nitric oxide synthetase (nNOS), the main source of NO production in neurons, in PD patients when compared to controls [80]. More than one-third of the protein constituents of Lewy bodies are prone to undergo S-nitrosylation, highlighting the importance of RNS and downstream S-nitrosylation of proteins in controlling many essential PD-related phenotypes such as axonal degeneration, impairment of dopaminergic metabolism and mitochondrial dysfunction [81]. Additionally, impaired mitochondrial complex I has been widely documented in cellular and animal models of PD as well as in PD patients’ brain and has been shown to contribute to PD pathogenesis [82]. Interestingly, the inhibition of mitochondrial complex I subunits by S-nitrosylation or nitration has been demonstrated in dopaminergic cells in different models of PD, which highlight the contribution of these redox-PTMs to the neurodegenerative process in PD [83].

### 2.6. Carbonylation

Protein carbonylation is the addition of reactive carbonyl groups (-CO), such as aldehydes, ketones and lactams, to amino acid side chains of proteins, inducing structural changes that often result in lowered activity and impaired protein function associated with multiple detrimental effects on cellular physiology [84,85]. Protein carbonylation can occur through pathways such as metal-catalysed oxidation, direct oxidation by ROS or oxidized polyphenols, and reaction with lipid peroxidation-derived aldehydes or reducing sugars (i.e., glycation/glycoxidation) [84,86,87,88]. Additionally, carbonylation can also be catalysed enzymatically with lysine carbonylation of extracellular matrix proteins by lysyl oxidases, representing a well-known modification [89]. Carbonylation is widely used as a representative marker for overall oxidation, albeit indicating a more advanced, irreversible state of oxidative damage associated with metabolic and age-related diseases [17,84,90,91,92]. Carbonylation has been previously observed on carboxyl-terminal hydrolase L1 (UCH-L1), a deubiquitinating enzyme that can be mutated in familial forms of early-onset PD [93]. Interestingly, carbonylation of UCH-L1 leads to similar detrimental effects on UCH-L1 function (incl. an increased insolubility) as PD-associated mutations in UCH-L1; this highlights the importance of aberrant redox-PTMs like carbonylation in the pathophysiology of sporadic PD, the most common form of PD [94]. Suggesting that carbonylation of proteins may be an early marker of AD, a number of proteins are aberrantly carbonylated in the brain of people with mild cognitive impairment (MCI, considered as an early stage of AD) as well as advanced AD—in particular, a number of glycolytic enzymes (pyruvate kinase, creatine kinase, enolase, α-ATP synthase) [95]. Given that aberrant carbonylation of these enzymes is correlated with their decreased activity and a decreased glucose metabolism in the brain of people with MCI and AD (as determined by brain imaging), it has been suggested that protein carbonylation is an early contributing factor to cognitive decline and progression in AD [95].

## 3. Methods Used to Identify and Quantify Redox-PTMs of Proteins

Several methods have been developed to study redox-PTMs, ranging from analyses of the whole proteome or of specific proteins to the detection of global redox-PTMs at specific residues or of specific redox-PTMs within proteins [15,92,96]. Chromatographic, colorimetric and enzyme-linked immunosorbent assays (ELISAs) can generally be used to study global levels of specific redox-PTMs of proteins or redox-PTMs on specific proteins; however, mass spectrometry (MS) techniques are usually required to precisely determine the identity and levels of oxidized proteins and their specific modified residues in cells or tissue [15,96]. A summary of the various redox-PTMs and the main approaches for detection is presented in Table 1.

Global protein oxidation is often evaluated by measuring global levels of carbonylation in cells and tissue [15,96]. Special emphasis has been placed on carbonylation due to its biochemical nature and because various analytical techniques can be employed to detect and quantify protein-bound carbonyls. The reactivity of carbonyl groups with 2,4-dinitrophenylhydrazine (DNPH) and other aldehyde-modified probes enables detection of the modification via simple methods such as colorimetric detection or antibody-based approaches (e.g., carbonyl-focused western blotting—“oxy-blotting”—and ELISAs), thereby supporting the widespread use of carbonylation measurement as an indicator of overall protein oxidation [17,96].

Measuring 3-NT in biological samples has also been used over the years as a biomarker for nitrosative stress in the settings of human pathologies [97]. Similar to protein carbonyls, 3-NT is a stable modification, making it useful for analysis using various techniques, such as immunological methods, liquid chromatography coupled with ultraviolet-visible absorption, electrochemical and diode array detections [98]. Similarly, evaluation of advanced oxidation protein products (AOPP)—a group of irreversible oxidatively modified protein products containing dityrosine (di-Tyr), pentosidine, and carbonyl-containing protein products (reactive C=O)—is also often used as a generic marker of oxidation. AOPP are most frequently derived from plasma proteins and can be easily quantified by chromatographic, colorimetric and ELISA assays [99,100,101,102,103].

Methionine residues can be readily oxidized by ROS to methionine sulfoxide (MetO), which is a promising marker of oxidative stress. Conventional methods to assay MetO formation and reduction rely on chromatographic or MS procedures. However, the quantitative analysis of methionine oxidation on a proteome-wide scale has been hampered by technical limitations, as methionine is readily oxidized in vitro during sample preparation and analysis, and there is a lack of enrichment protocols for peptides containing oxidized methionine residues [104,105].

### 3.1. Detection of Cys Oxidation

**Figure 2:**
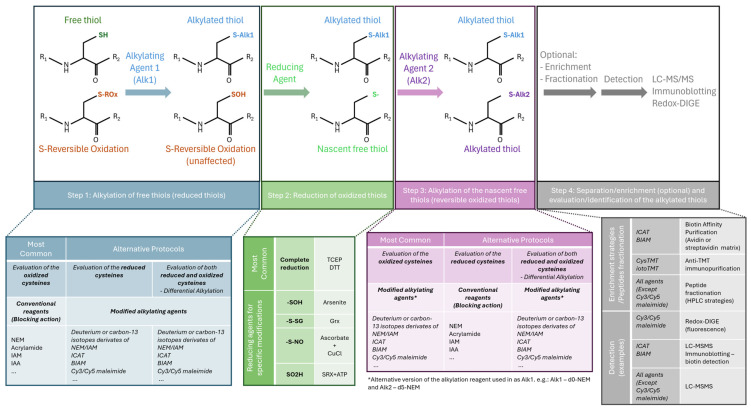
Overview of a generic tag-based reductive switch-labelling method for monitoring reversible protein thiol oxidations. In a conventional method for studying cysteine oxidation, the first step (in blue) involves alkylation of the free thiols, which can be achieved using a conventional alkylating reagent (such as N-ethylmaleimide (NEM), iodoacetamide (IAM), iodoacetate (IAA), etc.) to block free thiols (corresponding to the most common method) or a modified version of the alkylating agents if there is interest in studying the reduced Cys. During Step 2 (in green), reversibly oxidized thiols are reduced with a reducing agent that can be unspecific (such as dithiothreitol (DTT) or tris(2-carboxyethyl)phosphine (TCEP) for reducing all reversible Cys) or specific for a given modification (for example, arsenite for S-sulfenylation (S-OH), ascorbate for S-nitrosylation (S-NO), glutaredoxin for S-glutathionylation (S-SG)). The nascent thiols (reversible oxidized Cys) are then alkylated (Step 3 in purple) with an alkylating agent different from the one used in Step 1 or modified for use as probes or coupled with tags for purification/enrichment. The final step (Step 4 in grey) may involve separation (SDS-PAGE, HPLC) or enrichment (biotin affinity purification, antibody immunopurification) and identification of the alkylated Cys residues/alkylated proteins (typically by immunoblotting or MS analysis). Adapted from [35,36,37].

Given the importance of Cys oxidation in a range of biological functions and pathways, methods specifically targeting cysteines and their redox-PTMs have been developed [2,16,23,106]. These methods take advantage of the ability to irreversibly label the highly reactive thiolate ion present in Cys residues using specific compounds known as Cys alkylating agents; these agents can be modified to be used as isotope tags or as baits for enrichment techniques (Figure 2) [23,106]. Thus, most methods focus on the reversible oxidized forms of Cys, which can then be chemically reduced to their thiolate form for further alkylation—an approach commonly called a tag-based reductive switch-labelling method [107]. Briefly, the ‘switch assay’ is comprised of 4 main steps (Figure 2): (1) alkylation of free reduced Cys residues immediately after protein extraction, (2) reduction of reversibly oxidized Cys using either a general reducing agent such as dithiothreitol (DTT) or a modification-specific reductant such as ascorbate (to reduce sulfenylated Cys or nitrosothiols), or glutaredoxin (to reduce S-glutathionylated Cys), and (3) alkylation of nascent thiols with a distinct alkylating reagent, and finally, (4) detection using an appropriate method, typically an MS-based approach [2,23,108]. Variants of this protocol can be applied to focus on specific redox-PTMs; however, they all share the advantage of replacing labile oxidation-modified Cys with highly stable alkylated forms for subsequent analysis, while preserving the thiol oxidation state observed under physiological conditions [23,108]. Of note, if the modified alkylating agent is applied prior to sample reduction, the signal measured corresponds to the levels of reduced Cys; thus, it is inversely proportional to levels of oxidation. Conversely, if the modified alkylating agent is applied after reduction, the signal is directly correlated with the oxidation levels. Alternatively, both reduced and reversibly oxidized forms of Cys can be labelled simultaneously using different probes for each oxoform, usually a pair of light and heavy isotope-labelled compounds, allowing the monitorization of both reduced and reversibly oxidized oxoforms [23,109].

Different types of modified alkylating agents are available and can be used depending on the objectives, costs, and equipment available. Thanks to recent advances in MS, coverage of low-abundance species such as Cys-containing proteins has greatly increased [110]; however, comprehensive analysis of oxidized modified Cys in biological systems often requires a step of enrichment [111]. Sulfhydryl-specific biotinylated probes, such as biotin-Conjugated Iodoacetamide (BIAM) or biotin-HPDP ((N-[6-(Biotinamido)hexyl]-3′-(2′-pyridyldithio)-propionamide)), allow for subsequent streptavidin-pull down for enrichment of the biotin-labelled S-nitrosylated proteins followed by immunoblotting for the protein(s) of interest [38,107]. Fluorescent dye-labelled alkylating agents, usually cyanine dyes [112], can be combined with two-dimensional gel electrophoresis to allow for a simple inspection of spot patterns related to redox and protein-level changes [113]. However, this method lacks multiplexing capability (only two samples can be compared) and the capacity to identify the modified Cys/proteins [106,112]. Indeed, to identify and measure the levels of oxidized Cys residues, MS-based proteomics approaches are required to analyse the proteins/peptides containing labelled Cys [23,109].

In these methods, a comparative analysis between samples is performed by calculating the ratio of intensities of the Cys-containing peptides between samples and a control group. Of note, because irreversible Cys oxidations are stable, they typically cannot be labelled using the method described above (as the switch approach relies on the reduction of the redox-PTMs of interest). These irreversible redox-PTMs usually require the use of a targeted MS method, such as Multiple Reaction Monitoring (MRM) or Parallel Reaction Monitoring (PRM), with prior knowledge of which proteins and residues are modified and extensive method development [23,37].

Given the low abundance of Cys residues, redox proteomics assays often rely on the use of isotope tagging of Cys, such as the biotinylated iodoacetamide-based isotope-coded affinity tag (ICAT [114]) and Cys tandem mass tag (such as the reversible isobaric Cys-reactive tandem mass tag (cysTMT) [115] and the improved Cys-reactive TMT reagent version, the irreversible isobaric iodoacetyl Cys-reactive tandem mass tag (iodoTMT) [107]), followed by the enrichment of the alkylated Cys-containing peptides. Of note, unlike the use of other isotope-coded Cys-reactive tags, which are limited to pair-wise comparisons, the newest versions of isobaric TMT tags allow multiplexing up to six samples, taking advantage of the six channels of reporter ions, and the enrichment of the cysTMT-tagged peptides is facilitated by the accompanying anti-TMT resin, which proves to be more specific than a biotin-avidin-based capture [107].

Although these enrichment-dependent strategies result in an increase in the coverage of potential redox-sensitive Cys residues and an increase in sensitivity [37], the enrichment steps impede the determination of potential changes in protein abundance [106]. To overcome this limitation, some approaches have been developed to simultaneously analyse Cys oxidation and protein abundance, with CysTMTRAQ [106] and GELSILOX [116] being the first methods developed. However, CysTMTRAQ has a high cost due to the combination of two types of isobaric tagging (cysTMT for redox changes and iTRAQ for total level changes) [106], and the GELSILOX approach is based on the use of ^18^O enzymatic labelling of peptides, a labour-intensive and time-consuming procedure prone to errors and limited to the direct comparison of two samples [116]. To overcome these limitations, new methods have emerged, such as the Simultaneous Protein Expression and Redox (SPEAR) method, which involves the use of isotopically labelled alkylating agents (heavy and light forms of N-ethylmaleimide (NEM)) with online peptide fractionation and a high-resolution MS system [117], which allow the conventional label-free quantification of the non-Cys-containing peptides. In addition, other methods take advantage of advanced data-independent acquisition (DIA) methods, such as oxSWATH [113], a method based on the use of a differential alkylation pipeline using a pair of commonly available non-modified alkylating reagents in combination with sequential window acquisition of all theoretical fragment ion spectra mass spectrometry (SWATH-MS). More recently, CysQuant [118] involved the use of isotopically labelled alkylating agents combined with a DIA-acquisition method and a library-free deep neural network-based data analysis [119]. While oxSWATH may be a cheaper approach (as non-modified alkylating agents are used), it requires a higher amount of protein samples than SPEAR and CysQuant.

Additional approaches are currently being developed and validated, for instance, methods that focus on specific Cys modifications that have so far been neglected in previous assays, such as disulfide bonds [120] or those that directly target specific redox-PTMs using dimedone (5,5-dimethyl-1,3-cyclohexanedione) and its derivatives for the detection of sulfenic acid. Other examples are the use of antibodies on both Cys sulfinic and sulfonic acids or the use of reductive ligation of triarylphosphine derivatives and nitrosylated thiols. In addition, a combination of chemoproteomics probes with proximity-labelling methods has been developed to provide insights into subcellular Cys oxidation [2,121]. These new methods will help shed light on the functional role of specific redox-PTMs of Cys, such as how Cys oxidation affects the structure and interactome of proteins, as well as how redox-PTMs are spatially and temporally regulated in cells.

### 3.2. Methodological Considerations and Limitations

Selecting the most appropriate method to address a specific biological question is critical as it can significantly influence the outcomes of the study of redox-PTMs. When designing an experimental plan to study redox-PTMs, several factors need to be considered, including the type of samples and redox-PTMs investigated, whether an enrichment method is required or not, the number of samples to be compared, costs, quantification method (label-free, DIA, or label-based quantification), and the type of instrument available. Although MS has proven to be the most accurate and informative technique, in practice, method selection is often influenced by the equipment available, leading to a more generalized use of spectrophotometric methods or immunoassays, as they are often easier to access in most laboratories. However, while these methods may serve as convenient alternatives for assessing overall protein oxidation, they provide limited information and resolution as they tend to lack specificity and detect a wide range of oxidative species simultaneously without identifying the oxidized proteins, the specific redox-PTMs, or affected residues.

Regardless of the downstream approach used, one key challenge when analysing protein oxidation stems from its inherent instability due to its dynamic nature. Oxidized Cys residues can easily undergo reduction by other thiols or further oxidation [122,123]. Consequently, sample preparation plays a pivotal role in redox proteomics studies, as it is essential to preserve biological modifications while avoiding the introduction of artefacts [15]. Experimental factors that can alter the redox status of proteins include the lysis buffer-reducing agents, such as dithiothreitol (DTT) and mercaptoethanol, used when processing samples [1,124]. Moreover, atmospheric oxygen and free metal ions can induce further oxidation of samples. To prevent changes in the biological oxidation pattern or the formation of artefacts, samples should be prepared as quickly as possible, using procedures that promote the stabilization of reduced and oxidized residues with alkylating agents that prevent the oxidation of free thiol groups or derivatization reagents that modify the desired oxidative form, respectively [125]. Additionally, samples are often enzymatically digested prior to bottom-up MS, using common approaches such as gel electrophoresis and digestion, or digestion in solution, with these methods further exposing samples to atmospheric oxygen that can lead to the addition of artefactual oxidation, for example, S-thiolation of Cys residues, which may subsequently alter the results [96,126].

Another challenging aspect of redox proteomics analysis is the low prevalence of redox-PTMs in biological systems. To overcome this limitation, specific redox-PTMs from complex samples can be better detected and measured using alkylating agents combined with enrichment methods [96]. As mentioned above, numerous targeted MS methods utilizing tags and enrichment strategies have been developed to help facilitate the identification and quantification of redox-PTMs [127]. They each possess their own limitations with regard to the specificity of reagents and protocols required for each method, as well as their sensitivity in quantifying redox-PTM modified residues. To tackle some of these limitations, more complex protocols have been developed [127]. For example, a biorthogonal cleavable linker and switch technique to identify Cys residues (Cys-BOOST) has been recently developed, and this approach is more sensitive and specific in comparison to iodoTMT; however, Cys-BOOST requires a ten-step protocol as opposed to a five-step protocol for iodoTMT [128,129]. While increasing sensitivity and specificity, more complex and extensive protocols also increase the risk of technical errors and variability due to sample preparation.

Many of the redox-PTMs approaches described above have been developed and successfully used on cells in culture, where redox-PTMs can be artificially modulated with specific treatments and reasonably well-controlled. However, most of the methods currently available to detect redox-PTMs remain impractical for routine clinical use due to their complex methodologies and/or lack of automation and the risk of introducing artefactual redox-PTMs [102,130]. Furthermore, widely used methods of fixation and preservation of clinical tissue like formalin-fixation and paraffin-embedding can affect results obtained regarding redox-PTMs signatures.

In summary, the study of Cys residue oxidation has been dominated by MS methods focused on relative comparison between samples. Although several alternative methods have been developed, the sensitivity, specificity, and precision of these approaches generally depend on two aspects: (i) the MS instrument used (with the most recent high-resolution mass spectrometers providing better results), and (ii) the use of Cys/redox-PTMs enrichment or peptide prefractionation prior to the MS analysis. In general, the sensitivity of the methods increases if Cys enrichment or exhaustive peptide fractionation is performed. However, Cys enrichment may not accurately capture the native oxidized state of proteins under physiological conditions and will lose the information regarding protein expression levels necessary for the normalization of Cys oxidation levels, which may lead to potential bias [37].

Additionally, almost all the methods mentioned above, as well as other variants, share the common feature of tagging free thiols with modified alkylating reagents. In some cases, these modifications create large molecular compounds with limited access to react with Cys residues buried in native conditions [131]. Therefore, in most cases, only the most accessible Cys residues are considered in these types of studies. Furthermore, because these methods depend on the capacity to reduce Cys oxidation, they generally do not allow for the analysis of irreversible Cys oxidations, and in some cases, cannot distinguish the different types of Cys oxidation (see Table 1 for details).

As mentioned, most of these methods can be used for screening analyses to identify potential biomarkers. However, due to the complex sample processing and the requirements of advanced technical expertise, those methods are not commonly used in clinical practices (discussed in Section 6). Nevertheless, the methods mentioned above can help identify potential biomarker candidates for which MS-targeted approaches, such as oxMRM [132], or antibody-based approaches can be designed for both validation purposes and routine analysis [133].

## 4. Structural and Functional Effects of Redox-PTMs: Computational Modelling Contribution to Improve Mechanistic Understanding

Depending on the localisation and nature of the residue modified, redox-PTMs can differentially impact protein structure and function. For instance, redox-PTMs of methionine residues often impact protein structure and folding [134], while redox-PTMs of Cys residues usually affect catalytic activity and function of proteins because Cys residues are frequently present in protein active sites (up to 22% in human proteins) [10,18,20,21,135]. As discussed above, the study of redox-PTMs is relevant to understanding both health and disease. On the one hand, in physiological conditions, many redox-PTMs targeting Cys residues are reversible and act as a redox molecular switch through participation in intra- and inter-cellular signalling pathways [10]; on the other hand, Cys modified by irreversible redox-PTMs are often indicative of oxidative damage and can lead to protein misfolding, aggregation and dysfunction [2,136].

**Figure 3:**
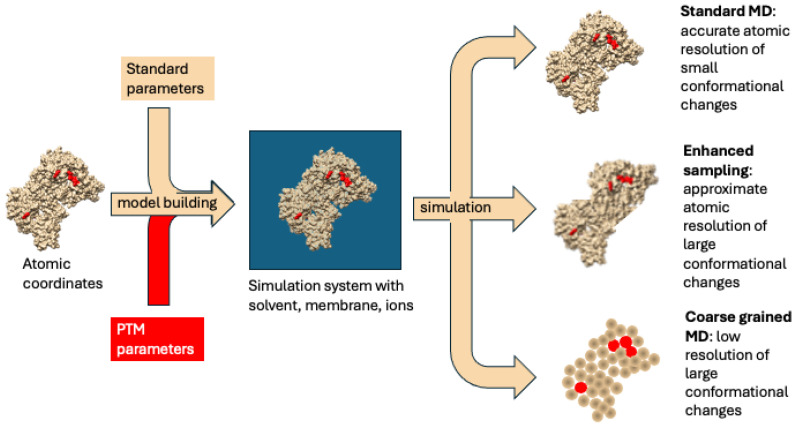
Essential steps in a molecular dynamics simulation workflow. Knowledge of an initial molecular structure is combined with established simulation parameters and—frequently—bespoke additional parameters to represent the PTM to produce a computational “system” that also represents the biologically relevant environment. From there, a variety of methods of molecular dynamics simulations may be employed—see text for details.

A variety of experimental methods can be used to explore how redox-PTMs affect protein function, including, but not limited to, the generation of proteins carrying point mutations of residues identified to be modified by redox-PTMs, artificial modulation of redox-PTMs in proteins with reagents such as redox donors (e.g., nitric oxide donors such as SNOC, GNSO, etc.) [137,138,139]. Techniques like nuclear magnetic resonance (NMR) spectroscopy or downstream functional assays can uncover the effect of redox-PTMs on protein structure [140]. These methods collectively share the capacity to assess the function of specific redox-PTMs on a particular biological process but typically fall short of providing an explanation of how the atomic-level alterations in protein structure that accompany redox-PTM result in changes in the function of modified proteins. Computational modelling approaches such as molecular dynamics (MD) simulations can fill this gap (Figure 3).

### 4.1. Molecular Dynamics of Protein Conformation Changes

MD simulations typically involve modelling the system of interest—atoms and bonds—as a collection of “balls and springs” whose structure and movements can be predicted using basic classical physical laws of motion [141,142]. Despite its apparent simplicity, this approximation has proved to be a powerful tool for visualizing the dynamics of proteins at the atomic level. Many biological processes involving PTMs involve some changes in protein function. Since an alteration in protein function is nearly always the consequence of some changes in its structure, flexibility, or molecular interactions, by correlating computationally predicted modulations in protein structure and dynamics produced by PTMs with experimentally observed changes in function, it is possible to decipher the molecular mechanism underlying the PTMs’ biological effect [142].

Because the MD simulation process is extremely computationally intensive, their scope and performance have increased steadily over the last 20 years as computers have become more and more powerful. Despite the improvement, the time scale for standard MD (sMD) simulations is still limited to the microsecond regime. As a result, many biological processes that happen over longer time periods cannot be directly studied using sMD simulations. Fortunately, by enhancing the sampling and simplifying the system, this limitation can be addressed effectively [137]. Enhanced sampling algorithms include targeted MD [143], which artificially steers a protein between conformations; metadynamics [144], which permits the free energy landscape of a protein’s conformational space to be “flattened” out by adding a history-dependent bias energy [144]; accelerated MD (aMD), which diminishes the energy barrier between conformational states [145]; along with the simplifying approach of coarse-grained modelling, which represents a system at a lower resolution where multiple atoms are represented by a single ‘bead’.

### 4.2. Molecular Dynamics to Explore the Effects of PTMs on Protein Conformation

There are many examples of studies where MD simulation technologies have helped understand the effect of various PTMs, including phosphorylation and glycosylation, on the structure of a target protein; however, there is a lack of literature regarding the use of MD simulations in the context of redox-PTMs. We will briefly review how MD simulations have been successfully used in the context of phosphorylation and glycosylation, highlighting their capacity and limitations, and will explore how similar approaches could be applied to the study of redox-PTMs in the future.

MD has, for example, provided a mechanistic hypothesis for how phosphorylation activates Src kinase [146]. The energetics and pathway of the transition from the inactive to the active state of Src were elucidated using an enhanced sampling technique (targeted MD) showing that the phosphorylation at a specific residue in Src and its interaction with ADP were the prerequisites for sampling the conformation corresponding to functional activation within biological timescales [146]. More recent advances in MD techniques have permitted the exploration of more complex choreographies of kinase activation at the atomic level. For example, metadynamics has enabled the exploration of the free energy landscape of another kinase, p38α, thereby quantifying the stability of different protein conformational states, and shedding light on the precise kinase activation mechanisms [147].

The study of large-scale conformational changes, such as the protein folding/unfolding equilibria that can be influenced by glycosylation, can stretch current atomistic simulation methods to their limit. To overcome this, an alternative approach, the coarse-grained method that simplifies the simulated protein by considering residues as the basic units instead of the atom as in sMD, can be used, mitigating computational costs and enhancing efficiency. For example, in silico studies have been able to decipher the folding of 63 engineered variants of the Src homology 3 (SH3) domain (a protein domain with roles in substrate recognition, membrane localization and regulation of kinase activity of proteins) that had been glycosylated with different numbers of conjugated polysaccharide chains at different sites on the protein’s surface [139]. Each amino acid and each sugar moiety were modelled as a single particle, greatly reducing the computational cost of the simulations. This approach revealed that glycosylation of the SH3 domain seems to promote folding because it destabilizes the unfolded state conformation while exhibiting minimal impact on the stability of the folded state. Extensive atomistic (not coarse-grained) molecular dynamics simulations on the epidermal growth factor receptor (EGFR) embedded in a model membrane environment uncovered the influence of glycosylation on EGFR, revealing a conformational rearrangement within the ligand-binding domains with the 2E9 ligand following N-glycosylation, which was consistent with experimental observations [148]. Together, these studies have demonstrated the power of using MD simulations to understand how PTMs can modify the structure of proteins, locally or through large conformational changes that impact protein activity and function.

Integration of experimental ‘wet lab’ and computational methods has been shown to be a powerful approach to uncovering how PTMs can affect protein structure and function. For instance, in the context of phosphorylation, multiple experimental techniques have identified the significance of Cys60-Cys77 disulfide bond formation in the activation of Akt2 kinase and subsequent recruitment to the plasma membrane [138]. These experimental findings were then combined with sMD simulations to better understand the mechanism of the activation process. sMD simulations showed that the disulfide bond enhanced the binding affinity of Akt2 to PIP3 by stabilizing its active site. Through the formation of the PIP3-Akt2 complex, Akt2 is then able to phosphorylate downstream substrates. However, these sMD simulations failed to offer insights into the process of plasma membrane recruitment. Also identified by experimental techniques, phosphorylation at Ser382 has been shown to activate lysine-deficient protein kinase 1 (WNK1), with its recruitment to vesicular structures being regulated by hyperosmotic stress [149]. sMD provided a hypothesis for the activation mechanism that the phosphorylation induces local stability within the activation loop but again provided little knowledge of its localization [150]. The two examples together illustrate the limitations of the current power of MD simulation methods, i.e., while relatively small-scale spatial changes can be simulated, the far larger-scale motions involved in plasma membrane recruitment remain out of reach.

### 4.3. Current Challenges in Molecular Dynamics Applied to Redox-PTMs

The challenge in conducting MD simulations of redox-PTMs lies in the fact that, unlike phosphorylation or glycosylation mentioned above, most redox-PTMs are not included in the force field libraries of commonly used MD simulation programs. The force field describes the interactions between atoms, and therefore, its presence is compulsory for the computation of MD (Figure 3). Generating reliable parameters for “unusual” molecular fragments is a complicated process, not least because it requires a ground truth—relevant experimental data—for the parameter refinement and validation process. The complexities involved in generating and analyzing data for many PTMs, as discussed earlier, pose significant challenges to progress in this field. However, despite these challenges, advancements have been made. For instance, to run MD simulations of S-nitrosylated (SNO) Cys, Han et al. [151] and Petrov et al. [152] separately generated the forcefield of SNO-Cys. With the force field, MD simulations of SNO modification in human serine racemase (hSR) were performed in parallel to experimental work utilizing specific point mutation, fluorescence spectroscopy and MS [153]. Initial experimental work demonstrated that ATP-bound hSR had a racemase activity, while S-nitrosylation of its Cys113 inhibited this activity. Subsequent 1 μs-long sMD simulations uncovered the precise underlying inhibitory mechanism, showing that the binding of ATP encouraged hSR to adopt a closed active conformation, and the SNO-Cys113 PTM changed the conformation towards a more open and less-active conformation. MD has also been successfully used to explore the structural and functional effects of S-nitrosylation in TNF Receptor Associated Protein 1 (TRAP1) [154]. Western blotting revealed that SNO favored the formation of a disulfide bond in TRAP1. Metadynamics simulations of SNO-Cys501 in TRAP1 revealed that S-nitrosylation induced a conformational rearrangement in the proximal Cys527 and favoured conformations bringing it close to Cys501, providing a structure-based explanation for the experimental observation. In another study, the impact of disulfide bonds on the structure of human serum albumin (HSA) was investigated through 70 ns-long sMD simulations in various combinations of oxidized and reduced states [155]. Because the only redox-PTM that was required to be simulated in this case was the very well-established disulfide bridge, force field parameterization issues were conveniently side-stepped. These simulations showed that disulfide bonds of Cys168-Cys177 and Cys278-Cys289 significantly contribute to the structural stability of HSA, whereas those involving Cys200-Cys246 and Cys461-Cys477 were found to have a minimal impact on HSA stability. Such information on key disulfide bonds in HSA can then provide guidance for experimentally engineering changes in the conformation and function of HSA. Therefore, despite the issues associated with the development of a reliable force field, there is good evidence that the methodologies that have been developed for running MD simulations of various PTMs such as phosphorylation and glycosylation have the potential to be successfully employed in the context of proteins modified by redox-PTMs.

## 5. Redox-PTMs in the Pathophysiology of NDDs

### 5.1. Redox-PTMs and Their Crosstalk in NDDs

PTMs represent an important way to increase the diversity of protein structures and functions through expansion of the range of adopted protein conformations and physicochemical properties of amino acids [156,157]. The PTMs-driven diversification allows a fine-tuned regulation of protein functions that can potentially impact numerous cellular biochemical and physiological processes, and disturbances in PTMs homeostasis have been associated with pathological conditions such as NDDs [156,158]. Importantly, PTMs can occur simultaneously, cooperating (both in positive and negative manners) to determine protein structure, localization and function [157]. Redox-PTMs, along with other non-redox-specific PTMs, such as phosphorylation, ubiquitination or SUMOylation, collectively play significant roles in regulating the activity of many redox-sensitive proteins. An example of this is the regulation of Prdx2, a key redox-sensitive protein, whose activity can be modulated by multiple redox-PTMs (as referred to in Section 2.1), as mentioned above, as well as non-redox-PTMs [33]. This regulation is essential for facilitating rapid cellular adaptation to various challenges and maintaining homeostasis; however, the precise underlying mechanisms of these processes remain unclear [58].

Due to the methodological constraints mentioned above, it remains challenging to determine PTMs co-occurrence, and therefore, the contribution of crosstalk between PTMs on protein conformation and how PTMs influence each other has remained poorly explored. However, recent technological advances, particularly in computational simulations, have facilitated the development of new strategies to study this complex interplay of PTMs [158]. In addition, new MS-based methodologies, coined PTMomics, have been developed in recent years to identify multiple PTMs simultaneously, including phosphorylation, cysteine modifications, and N-linked glycosylation [159,160]. For instance, this approach has successfully been used in the context of PD neurons in culture, uncovering the role of various PTMs in the pathophysiology of PD [161].

**Figure 4:**
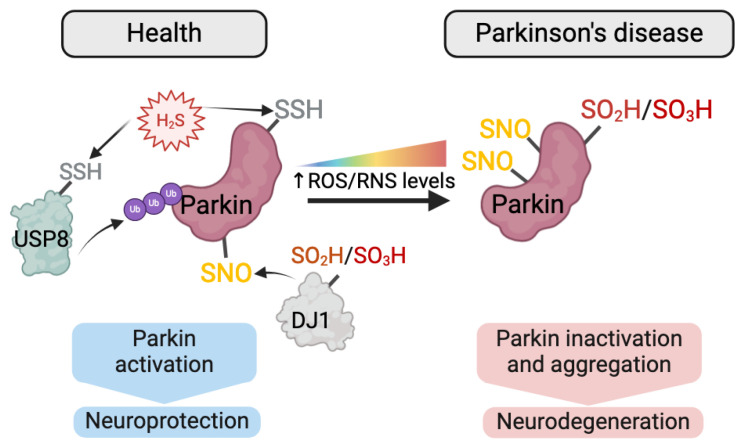
Crosstalk between redox-PTMs in the regulation of Parkin. The E3 ubiquitin ligase Parkin is post-translationally modified by multiple redox-PTMs that regulate its activity in a coordinated manner. Depicted are some examples of the complex interplay of redox-PTMs-driven regulation of Parkin activity. In physiological conditions, Parkin participates in the maintenance of the mitochondrial network homeostasis through the clearance of defective mitochondria by mitophagy. Mitophagy was shown to be promoted by increased S-sulfhydration of the deubiquitinase ubiquitin-specific peptidase 8 (USP8) [186], which in turn enhanced Parkin deubiquitination and recruitment to damaged mitochondria [186]. Additionally, direct hydrogen sulfide (H_2_S)-induced Parkin S-sulfhydration leads to the promotion of its activity, increased mitophagy and consequent prevention of protein aggregation, enhancing neuroprotection. However, under increasing levels of reactive oxidative species and reactive nitrosative species (ROS/RNS), such as in NDDs, there is an increase in aberrant S-nitrosylation of Parkin and a concomitant decrease in Parkin’s S-sulfhydration that results in an overall decrease in Parkin enzymatic activity. This decrease in turn leads to impaired clearance of misfolded proteins and subsequent accumulation of toxic oligomeric species as well as impaired mitophagy, with consequent neurodegeneration [177]. Additionally, S-sulfinylation and S-sulfonylation of Parkin are associated with its aggregation [178]. Conversely, S-sulfinylation and S-sulfonylation of DJ-1, which lead to its inactivation [22,171,174], modulates Parkin S-nitrosylation, enhancing its activity and altering mitochondrial quality control with a predicted impact on PD pathogenesis [171,179]. Adapted from [47]. Figure created with BioRender.

Highlighting the importance of intra-protein PTMs crosstalk, numerous NDDs-associated proteins have been shown to be modified not only by one type of PTM but by a combination of different redox-PTMs and non-redox-PTMs. These PTMs can sometimes exert opposing effects on the regulation of protein function. For instance, the activity of tyrosine hydroxylase (TH), the rate-limiting enzyme in dopamine synthesis (and other catecholamines) is regulated by multiple PTMs, including phosphorylation, S-nitrosylation, S-glutathionylation and nitration [162,163,164,165]. While phosphorylation and S-nitrosylation increase its enzymatic activity, S-glutathionylation and nitration inhibit it [162,163,164,165]. Similarly, Parkin, an E3 ubiquitin ligase and PD-linked protein, possesses reactive Cys residues that are susceptible to multiple redox-PTMs. S-nitrosylation of Parkin leads to changes in its E3 ubiquitin ligase activity while its sulfhydration on residues Cys95, Cys59 and Cys182 increases its enzyme activity [164,166] (Figure 4). In addition, redox-PTMs may affect other redox-PTMs within a protein. For instance, it has been shown that S-nitrosylation in the ALS/FTD-associated TAR DNA-binding protein 43 (TDP-43) facilitates the formation of intra-disulfide bonds in TDP-43, which triggers TDP-43 mislocalisation and aggregation observed in ALS/FTD [167].

Different redox-PTMs can also compete for the same residues in a given protein, which further increases the complexity of the intra-protein PTMs crosstalk. For instance, superoxide dismutase (SOD1), a protein associated with motor neuron disease ALS, can be modified by numerous non-redox-PTMs and redox-PTMs such as S-glutathionylation [168] or sulfenic acid on its Cys111 residue [169]. S-glutathionylation destabilizes SOD1 and initiates SOD1 aggregation while sulfenic acid triggers its fibrillation [170]; therefore, both redox-PTMs trigger different downstream molecular pathways but both contribute to SOD1 aggregation observed in ALS. However, it remains to uncover how S-glutathionylation and sulfenic acid compete for the same Cys111 residue, and how they interact with other non-redox-PTMs in SOD1 to regulate SOD1 folding and aggregation.

In addition, redox-PTMs in one protein can influence redox-PTMs in another protein (i.e., inter-protein redox-PTMs crosstalk); however, this important mechanism is still poorly understood. For instance, deglycase (DJ-1), a protein associated with early-onset PD [26,171,172], can be modified by various redox-PTMs, which modulate its activity and can, in turn, influence redox-PTMs in Parkin and α-synuclein (α-syn)—two key players in PD pathology [173] (Figure 4). Oxidation of DJ-1 at Cys106, a critical residue for DJ-1 redox-regulation, leads to sufinic, sulfonic and sulfinic acids [22,171,174], which control DJ-1 intracellular localization and enhance its effect in neuroprotection [171,175]. Additionally, sulfinic acid-modified DJ-1 exhibits anti-aggregation properties against α-syn, preventing its fibrillation [176]. An impaired sulfhydration and a concomitant increase in aberrant S-nitrosylation in Parkin lead to its inactivation and impaired clearance of misfolded proteins, as reported in postmortem brains of PD patients [177]. In addition, S-sulfinylation and S-sulfonylation alter Parkin E3 ubiquitin ligase activity and are associated with its subsequent aggregation [178]. Interestingly, the inactivation of DJ-1 has been suggested to modulate Parkin S-nitrosylation, thus enhancing its activity and altering mitochondrial quality control with predicted impact in PD pathogenesis [179] (Figure 4). Inter-protein redox-PTMs crosstalk also regulates another key function of Parkin as a regulator of mitophagy that clears dysfunctional mitochondria. Mitochondrial dynamics, including the processes of fission, fusion and mitophagy, represent a key quality control mechanism that regulates the homeostasis of the neuronal mitochondrial network [180]. Mitochondrial dynamics have been suggested to be regulated by ROS and redox-PTMs of proteins that modulate mitochondrial dynamics, such as Parkin [181,182]. For instance, *S*-glutathionylation regulates mitofusins (Mfn1/Mfn2), crucial players in the mitochondrial fusion process [183]. Dynamin-related protein 1 (Drp1), a protein involved in mitochondrial fission, is activated by S-nitrosylation [184], and high levels of S-nitrosylated Drp1 have been reported in postmortem brains of AD patients [185]. In addition, a recent study has shown that H_2_S promoted mitophagy by increasing S-sulfhydration of ubiquitin-specific peptidase 8 (USP8), which in turn enhanced Parkin deubiquitination and recruitment to damaged mitochondria (Figure 4) [186]. Another key factor in the regulation of mitophagy is the phosphatase and tensin homolog (PTEN)-induced kinase (PINK1), whose mutations are associated with autosomal recessive PD [187]. Mitochondrial damage-induced activation of PINK1 leads to Parkin phosphorylation-dependent activation and ubiquitin-dependent mitochondrial disposal through mitophagy [188].

Ageing is considered a major risk factor for all NDDs, which are commonly characterized by aggregation and accumulation of misfolded oxidized proteins. Interestingly, levels of S-sulfhydration have been reported to decrease with ageing, whereas an increase in S-sulfenylation and S-sulfonylation has also been observed [28,47,51]. The conjugation of these events was suggested to simultaneously increase the susceptibility of proteins to oxidative damage while also contributing to protein conformational changes that enhance protein aggregation. S-glutathionylation of free thiols has been demonstrated to prevent the formation of sulfinic and sulfonic acids [75]. Remarkably, it has been shown that the oxidation of Cys thiol groups to Cys sulfenylates promotes protein aggregation [28] and suggested that S-sulfhydration functions as a protective mechanism preventing protein aggregation. Moreover, S-sulfhydration generally occurs on the same Cys as S-nitrosylation, suggesting the two processes might be reciprocal, as exemplified above for Parkin. Therefore, the simultaneous alterations in these various redox-PTMs strongly suggest an intricate regulation of their crosstalk in redox signalling and maintenance of sulfhydryl homeostasis. Additionally, several PTMs have also been shown to be able to influence protein aggregation [189], with a paradigmatic example being α-syn aggregation in PD [190,191]. Together, the effects in protein redox regulation and modulation of protein aggregation highlight the relevance of redox-PTMs in age-related NDDs.

Although many redox-PTMs have been reported in several NDDs-related proteins and shown to contribute to NDDs pathophysiology, a comprehensive framework of redox-PTMs in terms of complexity and regulation is still incomplete. To explore redox-PTMs in ageing in vivo, a quantitative mapping of the Cys redox proteome in wild-type mouse at different ages, coined the Oximouse, has been recently described [192]. The Oximouse dataset quantified the percentage of reversible modifications of a wide range of Cys sites across several mouse tissues comparing young (16-week-old) and aged (80-week-old) mice in terms of redox modifications [192]. Importantly, in contrast with the predominant view that non-specific bulk protein oxidation increases with age, this study showed that tissues of aged animals displayed both age- and tissue-specific redox-regulated clusters. This study demonstrated that a reprogramming of the redox signalling landscape occurs in ageing in a tissue-specific manner. These data strongly argue in favour of an important role of redox-PTMs, as signalling effectors, in regulating age-related proteome remodelling both physiologically and in NDDs. Future developments in MS-based proteomics (to study multiple PTMs at once) and computational analysis will pave the way towards wider PTMs detection and interrogation of PTMs crosstalk [193,194,195,196].

### 5.2. Redox-PTMs as Biomarkers of NDDs

Because oxidative and nitrosative stresses are key contributors to the pathophysiology of NDDs and because they play a central role in modulating redox-PTMs of proteins, changes in the levels and identity of redox-PTMs of proteins have the potential to be useful indicators of disease onset and progression [197]. In particular, Cys residues and their oxidized forms have gained significance as potential biomarkers of protein oxidation in human diseases [19,198]. The potential of using redox-PTMs as biomarkers has been explored so far mainly in the context of cardiovascular and autoimmune diseases and remains to be investigated further in NDDs. Here, we review the lessons learned from using redox-PTMs as biomarkers in the context of other human diseases and discuss how this knowledge can be utilized in the future in the context of NDDs.

Biomarkers for disorders are valuable in clinical settings for distinguishing diseases with similar symptoms and clinical presentations and facilitating early detection [199]. For instance, in heart failure, the identification of irreversible Cys oxidation or carbonylation on cardiac-specific proteins like myofilaments, α-1-antitrypsin and fibrinogen in the blood of patients currently serves as a dependable prognostic biomarker [200], suggesting that redox-PTMs of circulating proteins could potentially serve as an indicator of heart failure. Such approach could be applied to diseases where oxidative stress contributes to disease progression, including NDDs. Of note, in addition to protein-based redox biomarkers, various oxidative stress biomarkers such as lipid peroxidation products (F2-isoprostanes (F2-isoPs)), and nucleic acid modifications (8-hydroxy-2′-deoxyguanosine) have been proposed as indicators of oxidative damage in heart failure [201]. For instance, F2-isoPs serve as reliable markers for monitoring oxidative stress due to their stability and high sensitivity. F2-isoPs can be detected in urine and serum samples [202]. A significant correlation has been discovered between increased levels of F2-isoPs in plasma and urine and the existence of coronary artery disease, as compared to individuals without the condition [203]. Specifically, this finding implies that the detection of F2-isoPs could be instrumental as a prognostic marker for heart failure. This is due to their strong correlation with the functional severity of the condition, positioning them as a proximate method to assess oxidative stress in combination with redox-PTMs of proteins. Identifying early disease onset remains challenging; however, there has been a promising rise in using novel biomarkers based on redox-PTMs of cardiac proteins for diagnosing and predicting heart failure [197].

Despite the current lack of redox-PTMs-based biomarkers for NDDs, there are potential biomarkers derived from redox-modified NDD-related proteins that could potentially be used in the future (Table 2).

Global levels of redox-PTMs can be used to monitor ROS and RNS levels, which are increased in NDDs. Because dysregulation of redox-PTMs of proteins contributes to the pathophysiology of many diseases, including NDDs [56], changes in their levels have been suggested as potential disease biomarkers [220,221,222]. For instance, the measurement of 3-NT levels is routinely used in biological samples derived from NDDs patients as a biomarker for nitrosative stress [97]. Oxidized albumin in the blood and CSF of patients has also been suggested to be a marker of systemic oxidative stress, as observed in patients with AD [204]. Although changes in global levels of redox-PTMs are unlikely to be specific to a particular NDD or to be used for diagnosis purpose, they could nevertheless be used to assess the effects of new drugs for NDDs in reducing ROS and RNS in the brains and hence be used as a proxy for assessing levels of oxidative and nitrosative stresses in the brain of people with NDDs.

Alternatively, redox-PTMs on proteins that are associated and/or that contribute to a given NDD can be used for diagnostic purposes. For instance, the accumulation of α-syn in insoluble aggregates is a key feature of PD and has potential as a PD biomarker [134,190]. In PD, α-syn is modified by diverse redox-PTMs that significantly influence α-syn aggregation and solubility and contribute to the formation of Lewy bodies [190,191,223]. α-syn has been shown to be modified by nitration in four tyrosine residues (Tyr39, Tyr125, Tyr133 and Tyr136) [214]. Importantly, nitrated α-syn is detected in PD biosamples suggesting nitrated α-syn as a potential biomarker for PD [224]. TDP-43 is another protein that could serve as a potential biomarker for NDDs. Indeed, specific PTMs have been identified on TDP-43, such as acetylation, phosphorylation, and Cys oxidation, and could serve as indicators of various NDDs, including AD and ALS, therefore holding promise as biomarkers for diagnostic purposes [225,226,227]. In line with this, excessively S-nitrosylated TDP-43 is detected in postmortem tissue of patients with ALS and FTD as compared to controls [167].

The presence or absence, or differential levels of redox-PTMs on specific proteins can be used to distinguish between various NDDs that may present with similar clinical features. For instance, PRDX2 is excessively S-nitrosylated in postmortem tissue of PD patients but not in AD patients, suggesting SNO-PRDX2 as a biomarker to differentiate between AD and PD [33]. The position of specific redox-PTMs in NDDs-associated proteins can also be considered a diagnostic tool. For instance, nitration at either Tyrosine residues 18 or 29 in the microtubule-associated protein (Tau) allows to distinguish between AD and other Tauopathies, including corticobasal degeneration, progressive supranuclear palsy and Pick’s disease, that present Tau aggregation and overlapping clinical features [228].

## 6. Future Directions

### 6.1. Redox-PTMs of Disease-Relevant Proteins to Diagnose NDDs

There is a growing number of biofluid- and imaging-based biomarkers that have been proposed to help with the diagnosis of NDDs [229]. For instance, AD diagnosis currently relies on a combination of clinical imaging and quantification of Aβ1-42, Tau protein and its phosphorylated form in the CSF of patients; however, these protein markers perform sub-optimally for early-stage sporadic AD. To tackle this, novel redox-modified biomarkers have been tested to help with the diagnosis of NDDs. For instance, Transthyretin (TTR), which is an abundant protein in the CSF, is present under unmodified and oxidized forms, and the relative changes in unmodified vs oxidized forms have been shown by Nano-LC-ESI MS/MS to successfully discriminate people with AD and MCI as compared to controls when combined with levels of amyloid β1-42, tTau, pTau [208]. This study also highlighted the importance of optimising sample handling to avoid introducing any artefactual redox-PTMs, in particular by controlling the duration of sample storage, experimental temperature, and the number of freeze–thaw cycles and developing a strict and reproducible sample preparation workflow [208]. After the initial phase of biomarker discovery in small- to medium-sized cohorts using untargeted proteomics, the biomarker validation phase is usually performed in larger independent cohorts. For instance, novel biomarkers to support the diagnosis of DLB (which can be clinically misdiagnosed as AD) have been initially identified using label-free proteomics in CSF on a small cohort; this led to the discovery of a set of six potential biomarkers, including neurosecretory protein VGF [230]. This was later validated using targeted proteomics in an independent cohort of patients with DLB and in a larger cohort that included patients with AD, PD, or FTD [230]. In addition, multiple orthogonal proteomics methods have to be employed to validate new diagnosis biomarkers; these methods include targeted LC-MS/MS proteomics, ELISA, Olink proximity extension assay, and SomaScan aptamer precipitation assay [230,231].

### 6.2. Redox-PTMs of Disease-Relevant Proteins to Follow Disease Progression

Given that ROS and RNS levels increase with NDDs progression, changes in redox-PTMs are expected over the course of the disease. For instance, an increase in the levels of S-glutathionylated GAPDH is observed in the blood of people living with AD and has been shown to be correlated with disease progression and severity [207]. Similarly, redox proteomics on postmortem brains from people on the AD continuum, from preclinical AD (PCAD), MCI, to AD, showed that nitration and carbonylation of proteins change with disease progression (reviewed in [232]): while no difference in global protein carbonyl or nitration is detected in PCAD compared to controls, an increase in protein carbonyl is observed in MCI compared to PCAD [232,233]. Similarly, in the context of PD, levels of nitrated α-syn in the serum of PD patients correlate with disease severity and worsened PD-related outcomes; this suggests that nitrated α-syn may be used to follow disease progression and as a prognostic biomarker for PD [234]. In line with these observations, it has been suggested that with the progression of NDDs, a progressive shift not only in the level but also in the nature of the redox-PTMs, from labile to potentially more stable and irreversible, would be detected on disease-relevant proteins [24]. Future longitudinal studies quantifying specific redox-PTMs on disease-relevant proteins at different stages of NDDs will be required to test this hypothesis. In recent longitudinal proteomics studies on the CSF and plasma of people with MCI, prodromal or symptomatic people carrying mutations in AD- or PD-associated genes showed that protein changes occur even decades before the onset of symptoms [235,236,237]. Although these studies focused on global protein levels and did not consider PTMs of proteins, they demonstrate the feasibility of such an approach to uncover protein changes at different stages of the disease and could be applied in the future to the study of redox-PTMs of proteins.

### 6.3. From Discovery of Redox-PTMs of Proteins to Implementation in the Clinic

Stable or irreversible redox-PTMs may represent more reliable biomarkers compared to transient and labile redox-PTMs because the latter are more prone to artefactual alterations during sample handling and remain challenging to quantify (as discussed in Section 3). However, stable or irreversible redox-PTMs may represent more advanced and later stages of the disease and therefore might not be suitable biomarkers to diagnose NDDs at prodromal or early stages of the disease.

MS-based proteomics is a powerful tool to discover and validate new biomarkers for NDDs; however, it is not routinely used in the clinic mainly due to costs, the need for highly specialized equipment, and complex sample preparation and analysis (as discussed in Section 3) [231]. Therefore, for biomarkers identified by MS-based proteomics to be routinely used in the clinic as diagnostic and prognostic tools, they would have to be detectable using relatively cheap, quick and easy approaches (e.g., Simoa, ELISA, chemoluminescence and electrochemiluminescence assays) [231]. The level of oxidation (unmodified vs. oxidised) and type of redox-PTMs of the biomarkers selected will have to be detectable within the selectivity and sensitivity range of such approaches. Importantly, to increase reliability and accuracy, it is expected that a panel of redox-PTMs on multiple disease-relevant proteins would have to be considered in parallel.

In addition, to be used clinically, redox-PTMs of proteins would have to be detectable in patients’ samples such as CSF or blood. CSF has been used extensively to investigate potential biomarkers for NDDs due to its proximity to the central nervous system [238]. Blood-based biomarkers would make sample collection less invasive and easier than CSF-based biomarkers; however, systemic oxidative stress present in the blood circulation of people living with NDDs has been suggested to affect redox-PTMs of proteins when proteins cross the blood–brain barrier [212]. Thus, redox-PTMs of proteins in the blood may not represent the oxidative status of the protein in the brain. Therefore, potential blood-based redox-PTMs biomarkers will also have to be validated in CSF and/or postmortem tissue. For instance, excessively nitrated ATP synthase F1 subunit alpha (ATP5F1A) is detected not only in postmortem tissue of AD patients [211] but also the blood cells of AD patients [212], suggesting nitrated ATP5F1A as a robust biomarker for AD. Interestingly, it has recently been shown that salivary protein profiling allows to distinguish PD and AD from controls, suggesting that proteins from saliva may be used in the future as another non-invasive and easily accessible source of biomarkers for NDDs diagnosis [239]; however, whether redox-PTMs would be preserved in salivary samples has yet to be explored. The quantification of redox-PTMs in bodily fluids, such as CSF or blood, remains technically challenging, as discussed in Section 3.2; therefore, strict methodological workflows would have to be developed to reduce technical artefacts being introduced [240]. It would be essential that methods be standardized and include reference materials, such as commercially available oxidized proteins, to ensure result consistency for comparison purposes [15]. Therefore, before such biomarkers can reach the clinic, important aspects will have to be addressed, including defining cut-offs for diagnosis thresholds.

## 7. Conclusions

Redox-PTMs of proteins are important and ubiquitous PTMs that regulate key cellular functions and contribute to brain physiology, ageing and NDDs. The precise mechanisms of redox-PTMs in regulating the conformation and function of proteins are yet to be fully understood and will rely on technical developments in MS-based proteomics methods and in silico strategies. Together, these new approaches will offer a promise not only to better understand the role played by redox-PTMs in the pathogenesis of NDDs but also to find potential biomarkers for these disorders. Indeed, because oxidative and nitrosative stress and associated redox-PTMs of proteins have been suggested to occur years before any neurodegeneration or symptoms, redox-PTMs have the potential to be used as biomarkers of NDDs in the prodromal phase of NDDs. Discovering such early biomarkers will be essential in providing more effective and timely therapeutic interventions to prevent the onset of NDDs or to slow down their progression.

## Figures and Tables

**Table 1:** Summary of the main redox-PTMs, residues affected, reversibility and generic detection approaches.

Residue Affected		Redox-PTM	Reversibility	Methods of Detection
Cysteine residues (C)		Reduced (Free) thiols (-S-H)	---	Colorimetric-based assays—Ellman’s assay Fluorescence-based assay—reaction with monobromobimane Tag-based reductive switch-labelling method (see Figure 2)
		Disulfide bonds (-S-S)	Reversible	Tag-based reductive switch-labelling method (see Figure 2) with a complete reductant agent **(no specific detection)** Molecular shift on SDS-PAGE
		S-sulfhydration/ Persulfidation (-S-SH)	Reversible	Tag-based reductive switch-labelling method (see Figure 2) with a complete reductant agent **(no specific detection)**
		S-glutathionylation (-S-SG)	Reversible	Tag-based reductive switch-labelling method (see Figure 2) with a complete reductant agent **(no specific detection)** or **glutaredoxin** reduction **(specific detection)**
		S-nitrosylation/ S-nitrosation (-S-NO)	Reversible	Tag-based reductive switch-labelling method (see Figure 2) with a complete reductant agent **(no specific detection)** or reduction with **ascorbate and copper chloride (specific detection)** Fluorescence-based assays—reductive reaction with triarylphosphine derivatives
		S-sulfenylation (-SOH)	Reversible	Tag-based reductive switch-labelling method (see Figure 2) with a complete reductant agent **(no specific detection)** or reduction with **arsenite (specific detection)** Reaction with dimedone—detection by: - Colorimetrical-based assays - MS - Antibody-based approach
		S-sulfinylation (-SO_2_H)	Reversible	Tag-based reductive switch-labelling method (see Figure 2) enzymatic reduction with **sulfiredoxin (specific detection)** Direct detection by MS Antibody-based approach
		S-sulfonylation (-SO_3_H)	Irreversible	Direct detection by MS Antibody-based approach
Other residues	Arg (R) Pro (P) Lys (K) Thr (T)	Carbonylation (C=O)	Irreversible	Reaction with DNPH and other aldehyde-modified probes—detection by: - Colorimetric detection - Antibody-based approaches (“oxy-blotting”—and ELISAs)
	Tyr (Y) Arg (R) Lys (K) Pro (P) Glu (E) Thr (T)	Advanced Oxidation Protein Products (AOPP): dityrosine (di-Tyr) pentosidine carbonyl-containing protein products (reactive C=O)	Irreversible	Chromatographic assays Colorimetric assays ELISA assays
	Tyr (Y)	Nitration (3-NO_2_-Tyr)	Irreversible	Antibody-based approaches Liquid chromatography coupled with ultraviolet-visible absorption, electrochemical and diode array detections
	Met (M)	Oxidation (Met=O)	Irreversible	Chromatographic assays MS methods

**Table 2:** Redox-PTMs of proteins as potential biomarkers for NDDs. Examples of redox-PTMs detected in human postmortem tissue, blood, cerebrospinal fluid (CSF) in Alzheimer’s disease (AD), Parkinson’s disease (PD), amyotrophic lateral sclerosis (ALS), frontotemporal dementia (FTD), dementia with Lewy bodies (DLB), multiple system atrophy (MSA), and multiple sclerosis (MS). SOD1: superoxide dismutase 1; GAPDH: Glyceraldehyde 3-phosphate dehydrogenase; GSK3β: Glycogen synthase kinase-3 beta; TTR: Transthyretin; deoxy-Hb: deoxy hemoglobin; CRYAB: Alpha-crystallin B; ENO1: Enolase 1; IL-18: Interleukin-18; α1-AAT: Alpha-1 antitrypsin; SOD2: superoxide dismutase 2; ALDOC: Aldolase C; VDAC2: Voltage-dependent anion-selective channel protein 2; TDP-43: TAR DNA-binding protein 43; ATP5F1A: ATP synthase F1 subunit alpha; CAT: catalase; VDAC: voltage-dependent anion-selective channel; α-Syn: α-synuclein; PRDX2: Peroxiredoxin 2; A1AT: Alpha-1 antitrypsin; FGG: Fibrinogen gamma chain; UCH-L1: Ubiquitin C-terminal hydrolase L1; 2-DE: 2-D gel electrophoresis; WB: Western blotting; IHC: immunohistochemistry; IP: immunoprecipitation; DNP/DNPH: 2,4-dinitrophenylhydrazine; 3-NT: 3-nitrotyrosine; PBMCs: peripheral blood mononuclear cells; LID-PRM: laser-induced dissociation-parallel reaction monitoring. ↑ indicates an increase in the levels of global redox-PTMs or specific redox-PTM-modified proteins; ↓ indicates decrease in the levels of specifically PTM-modified proteins.

Redox-PTMs	Disease	Biological Sample	Method(s) Used	Variation/Alteration	Refs.
Sulfenic acid, sulfinic acid and/or sulfonic acid	AD	CSF, blood	HPLC, LC-ESI-qTOF-MS	↑ reversible (disulphide bond) and irreversibly (SO_2_H) oxidized albumin, ↓ reduced (-SH) albumin (higher differences in CSF than blood)	[204]
	ALS	CSF	WB, ELISA	↑ SO_2_H/SO_3_H of wild-type SOD1 in sporadic ALS	[170]
	PD	Postmortem tissue	WB, IHC	↑ sulfonated Parkin in insoluble fractions in PD; ↑ Parkin in insoluble fraction (aggregation)	[178]
Disulfide bonds	AD	Postmortem tissue	Redox 2DE	↑ disulfide bonds in GAPDH in AD	[45]
S-sulfhydration	AD	Postmortem tissue	Dimedone-switch assay; antibody array-like approach	↓ global sulfhydration; ↓ sulfhydration of GSK3β (Tau kinase)	[55]
	PD	Postmortem tissue	Maleimide assay	↓ S-sulfhydration of Parkin	[177]
	MS/ALS/AD/PD	CSF	MALDI-TOF-MS	↑ global H_2_S-protein bound and ↑ TTR-H_2_S in MS (no significant increase in ALS, AD, PD as compared to controls)	[205]
S-glutathionylation	AD	Postmortem tissue	2DE; Oxyblots; MALDI-TOF	↑ S-glutathionlylation of deoxy-Hb, CRYAB, GAPDH, ENO1 in AD	[206]
	AD	blood	ELISA	↑ S-glutathionylated GAPDH in AD, and levels correlate with AD progression	[207]
	AD	CSF	IP of TTR and nanoLC-ESI-MS	↑ S-glutahionylated TTR in AD and MCI	[208]
	MS	CSF	nano-LC/ESI-MS	↑ S-glutahionylated proteins in MS patients during relapse incl. IL-18; α1-AAT	[209]
S-Nitrosylation	AD	Postmortem tissue	2D-Oxyblot; biotin switch; ESI-QTOF MS/MS; IHC against SNO-Cys	↑ global S-nitrosylation levels in AD. 45 S-nitrosylated proteins identified in AD incl. ↑ S-nitrosylation of SOD2, ALDOC, VDAC2 in AD	[210]
	FTD	Postmortem tissue	Biotin switch-WB	↑ S-nitrosylation and disulfide bonds in TDP-43 in FTD	[167]
	PD	Postmortem tissue	Biotin switch-WB	↑ S-nitrosylation of Parkin	[164,166]
	PD/AD	Postmortem tissue	Biotin switch-WB	↑ S-nitrosylation of PRDX2 in PD brains when compared to controls, but not in AD brains	[33]
Nitration	AD	Postmortem tissue	2DE; MALDI-TOF-MS	↑ nitration of proteins in AD incl. ATP5F1A, VDAC, GAPDH	[211]
	AD	Lymphocytes in blood	2DE; HPLC-ESI-MS/MS; Slot blot; IP/WB with anti-3NT antibody	↑ global 3-NT levels in AD; ↑ 3-NT in 10 proteins incl. ATP5F1A, CAT	[212]
	ALS	Postmortem tissue	IHC with anti-3-NT antibody	↑ Immunoreactivity for 3-NT-positive motor neurons in ALS	[213]
	PD/DLB/MSA	Postmortem tissue	ELISA; IHC; WB with anti-nitrated-α-Syn	↑ nitrated α-Syn in PD, DLB, MSA	[214]
Carbonylation	AD	plasma	2DE; WB with anti-DNP antibody; MALDI-TOF/MS	↑ carbonylation of seven proteins identified incl. A1AT, FGG precursor in AD	[215]
	AD	Plasma	HPLC, WB with anti-DNP antibody	↑ carbonylated protein levels in AD	[216]
	AD	CSF	2DE; Oxyblots; MALDI-ToF MS	↑ carbonylated protein levels in MCI and AD; 7 proteins identified with ↑ carbonylation, incl. APOE.	[217]
	AD	Postmortem tissue	2DE; MALDI-TOF/MS; HPLC-ESI/MS/MS	↑ carbonylated SOD1 in AD and PD.	[218]
	ALS	PBMCs in blood	DNPH assay	↑ carbonylated proteins in ALS	[219]
	PD/AD	Postmortem tissue	2DE; MALDI-TOF/MS; HPLC-ESI/MS/MS	↑ carbonylation, cysteine and methionine oxidation of UCH-L1	[93]
